# Decreased E2F2 Expression Correlates with Poor Prognosis and Immune Infiltrates in Patients with Colorectal Cancer

**DOI:** 10.7150/jca.61415

**Published:** 2022-01-01

**Authors:** Yuanyuan Shang, Yuanyuan Zhang, Jie Liu, Lin Chen, Xudong Yang, Zhe Zhu, Dan Li, Yewei Deng, Zhuqing Zhou, Bing Lu, Chuan-gang Fu

**Affiliations:** Department of Colorectal Surgery, Department of General Surgery, Shanghai East Hospital, Tongji University School of Medicine, Shanghai, China.

**Keywords:** E2F2, colorectal cancer, prognosis, immune infiltration, bioinformatics analysis, immunohistochemistry

## Abstract

Growing evidence has revealed that the E2F family of transcription factor 2 (E2F2) participates in the tumorigenesis and progression of various tumors, but its role in colorectal cancer (CRC) remains largely unknown. Herein, the aim of our study was to investigate the exact role of E2F2 in CRC. The expression levels of E2F2 in CRC were appraised based on the Tumor Immune Estimate Resource (TIMER), Oncomine, The Cancer Genome Atlas (TCGA), Gene Expression Omnibus (GEO) database. The results were further confirmed using CRC tumor tissues and normal controls by experimental assays including immunohistochemistry, qRT-PCR and western blot. The survival analysis of E2F2 in CRC was analyzed using PrognoScan database and TCGA data sets. In addition, the functional roles of E2F2 were examined by Gene Set Enrichment Analysis (GSEA) and immune infiltration analysis. Our results illustrated that E2F2 was significantly downregulated in CRC samples. The low E2F2 expression in CRC was prominently correlated with N, M stage and pathological stage. Decreased E2F2 expression had an unfavorable overall survivial (OS), disease free survival (DFS), disease specific survival (DSS) and progress free interval (PFI). Multivariate cox regression showed E2F2 could be an independent prognostic factors of OS in CRC. Receiver operating characteristic (ROC) analysis showed that E2F2 may serve as a potential diagnostic biomarker for CRC patients. GSEA disclosed that E2F2 was probably involved in several pathways, including ATR pathway, ATM signalling pathway, mismatch repair, base excision repair, homologous recomibination, Fanconi Anemia pathway, multicancer invasiveness signature, and cancer stem cells. Moreover, E2F2 was significantly correlated with the infiltration level of Th2, aDC, Th17, NK CD56dim, T helper and pDC cells. The current study demonstrates that decreased E2F2 expression is closely associated with poor prognosis and immune cell infiltration in CRC, which can be a promising independent prognostic biomarker and potential treatment target for CRC.

## Introduction

Colorectal cancer (CRC) remains the third most frequently diagnosed malignancy and the second leading cause of cancer-related mortality worldwide, accounting for ~10% of all detected cancer cases and ~9% of all cancer deaths 1, 2. Despite considerable efforts have been directed toward early diagnosis and multidisciplinary treatment in CRC management, including improved screening methods, surgical procedures, chemotherapy, radiotherapy, targeted biotherapy and immunotherapy, a significant portion of patients with CRC, especially patients with advanced-stage (stage III and IV) tumor, present with poor prognosis 3-5. The 5-year survival rate for patients with early-stage CRC is approximately 90%, whereas the survival rate drops to 13.1% once patients are diagnosed with advanced-stage CRC 6. Carcinogenesis and tumor progression of CRC is a complex multi-step, multi-factor process, which involves the development from normal epithelium to precursor adenomatous polyp and finally to carcinoma 7. Large-scale of studies have revealed that multiple genetic mutations, epigenetic alterations, and aberrant molecular signaling pathways are indispensable for cancer development and progression 8. Therefore, it is urgently required to investigate more detailed molecular mechanisms to identify early screening biomarkers and potential therapeutic targets in CRC.

E2F family of transcription factors, widely expressing in different tissues and organs in higher eukaryotes, are considered to play a pivotal role in cell cycle regulation, cell proliferation, differentiation, necrosis, DNA repairing, chromatin assembly and condensation and chromosome separation 9-11. Classically E2F family components are divided into two opposite subfamilies: activators of transcription (E2F1/2/3a) and transcriptional repressors (E2F3b/4/5/6/7/8). However, each family member exerts diverging roles and functions depending on the tissue and cell context 12, 13. E2F2, a member of E2F family, has emerged as an important modulator in several human malignancies, attracting rising attention as a potential predictive biomarker and targeted therapeutic gene in recent years. E2F2 can function as either a tumor promoter or suppressor depending on the environment. It was reported that E2F2, as an oncogene, is prominently upregulated in patients with ovarian cancer, and aberrant overexpression of E2F2 is obviously correlated with poor clinical outcomes based on multiple bioinformatics database 14. In glioblastoma multiforme, E2F2, as a direct target gene of Let-7b, is confirmed to promote the proliferation of glioma and glioma stem-like cells 15. In human melanoma cells, the NAMPT/E2F2/SIRT1 axis promotes proliferation and inhibits p53-dependent apoptosis 16. However, E2F2 can also serve as a tumor suppressor in many malignancies. Yu Gao et al. 17 reported that E2F2 expression is shown to be downregulated in clear cell renal cell carcinoma cancer tissues compared with normal tissues, and to reduce tumor proliferation and motility significantly. E2F2 has also been suggested as a specific tumor suppressor in Myc-induced T cell lymphomagenesis 18. However, the precise role of E2F2 in prognosis and biological function has not been well established in CRC.

In our present study, we comprehensively analyzed the correlation of E2F2 expression and clinical pathological characteristics of patients with CRC by using multiple publicly available databases and our clinical specimens. Next, we determined the diagnostic and prognostic value of E2F2 in CRC. Moreover, we investigated the possible pathogenic mechanism of E2F2 by performing gene-set enrichment analysis (GSEA) for high and low E2F2 expression group. Finally, since tumor microenvironment is an important participant of tumor initiation and progression, the relevance between E2F2 expression and immune cell infiltration of CRC was also discussed. This study not only highlights the importance of E2F2 in CRC, but also indicates the potential of E2F2 to serve as a prognostic biomarker and therapeutic target in CRC.

## Materials and methods

### TIMER database analysis

Tumor Immune Estimate Resource (TIMER, http://timer.cistrome.org) is an online database used for comprehensive analysis of tumor-infiltrating immune cells and different gene expression levels in different cancer types 19. We used the TIMER database for studying the differential expression of E2F2 between tumor and normal tissues across various cancer types.

### Oncomine database analysis

The oncomine database (https://www.oncomine.org/resource/main.html) is a database comprised of publicly available cancer microarray data and was used to identify E2F2 mRNA expression levels in different tumors 20. The thresholds for E2F2 were set as p < 1E-4, a 2-fold change and a top 10% of gene ranking. The data type was mRNA.

### RNA-sequencing data and bioinformatic analysis

Normalized RNA-seq data and corresponding clinicopathological information of 647 colonic (COAD) and rectal (READ) tumor tissues and 51 normal tissues were acquired from The Cancer Genome Atlas database (TCGA-COAD and TCGA-READ data sets). The data format of level 3 HTSeq-fragments per kilobase per million (FPKM) were downloaded. The main clinicopathological feature information of CRC patients was shown in **Table 1**. There were some patients with incomplete clinical information and the patients were only excluded when comparing the specific clinical factors that they lacked. In addition, to validate the E2F2 mRNA expression in patients with CRC, the raw gene profiles of GSE20916 21 and GSE9348 22 were downloaded from the Gene Expression Omnibus (GEO) database.

### Patients and clinical tissue samples

The paraffin-embedded tissues from a cohort of 102 stage I-III CRC patients with tumor tissues and 27 healthy controls with normal mucosa tissues were collected in Changhai Hospital, affiliated to Second Military Medical University (Shanghai, China) between 2001 and 2010. In addition, we obtained 5 pairs of primary CRC and corresponding adjacent normal tissues from the Department of colorectal surgery in Shanghai East Hospital (East Hospital Affiliated to Tongji University, Shanghai, China) in 2021. The adjacent normal tissues were extracted > 3 cm from the tumor margin, and were evaluated by microscope to exclude dysplastic cells. All of the CRC patients, without other malignant tumors, underwent curative surgical resection and were pathologically diagnosed as CRC. None of them were treated with neoadjuvant chemo-radiotherapy or other special treatment before surgery. Pathological staging was determined according to the American Joint Committee on Cancer classification system, 7^th^ edition, 2010. The general clinicopathological characteristics of these CRC patients are summarized in **Table 1**. All patients signed the written informed consents and this study was approved by the Institutional Review Board of Changhai Hospital and the Medical Ethics Committee in Shanghai East Hospital.

### Immunohistochemistry analysis

The 129 paraffin-embedded tissues were dewaxing and rehydrating, and then submerged into EDTA solution (1 mM, pH8), heated for 1 hour by steamer (about 95 °C), treated with 3% hydrogen peroxide, pre-incubated with 10% normal goat serum, and incubated with mouse anti-human E2F2 monoclonal antibody (sc-9967, Santa Cruz Biotechnology Inc., CA) overnight at 4 °C. Next, the treated sections were incubated with mouse lgG Fc binding protein (m-lgG Fc BP) conjugated to horseradish peroxidase (sc-525409, 1:100, Santa Cruz Biotechnology Inc., CA). The 3,3' di-amino-benzidine (DAB) solution was used as chromogen and Harris hematoxylin was used as counterstain. Finally, the slides were dehydrated and mounted.

For evaluation, staining localized in the cytoplasmic, nuclei and/or membranous was considered positive. Images were captured using a Leica microscope. Image-Pro Plus software (version 6.0, Media Cybernetics Inc. Rockville, MD, USA) was used to evaluate the area and the integrated optical density (IOD) value of the dyed region in the IHC section. The average optical density (AOD) of the digital image (magnification, x200) was adopted as representative E2F2 staining intensity. AOD = IOD/Area. The three randomly selected fields from each tissue section were captured in a blinded manner, and then the AOD value was calculated and subjected to statistical analysis.

### Western blot assay

Tissue proteins were extracted by RIPA lysis buffer (Biyuntian Biotechnology Co., Ltd., Shanghai, China) and quantitated. Equal amounts of tissue protein samples were separated on 10% SDS-polyacrylamide gel, and then transferred onto polyvinylidene fluoride (PVDF) membranes (Millipore, Eschborn, Germany). After blocked with 5% skim milk in tris-buffered saline tween (TBST) at room temperature for 2 h, the membranes were probed with the primary antibodies: mouse anti-human E2F2 monoclonal antibody (55 kDa; 1:1000) (sc-9967, Santa Cruz), rabbit anti-human GAPDH antibody (36 kDa; 1:1000) (sc-47724, Santa Cruz) at 4 °C overnight. After the membranes were incubated with secondary antibodies at room temperature for 1 h, an enhanced chemiluminescence detection system was used to detect the signals.

### Quantitative real-time polymerase chain reaction (RT-qPCR)

According to the manufacturer's instructions, total RNA was extracted from tissues by TRIzol Reagent (ThermoFisher, CA, USA). Reverse transcription to cDNA was carried out using PrimeScript™ RT reagent kit (TaKaRa). RT-qPCR was carried out in the Real-Time PCR System (Roche, Meylan, France) using the SYBR Premix Ex Taq™ (TaKaRa). The primer sequences were as follows: E2F2 forward primer, 5'-GAGCTCACTCAGACCCCAAG-3', and E2F2 reverse primer, 5'-AACAGGCTGAAGCCAAAAGA-3'. GAPDH forward primer, 5'-CATGAGAAG TATGACAACAGCCT‐3', and GAPDH reverse primer, 5'‐AGT CCTTCCACGATACCAAAGT-3'. The 2-∆∆Ct comparative method was applied to calculate the relative E2F2 mRNA expression.

### PrognoScan Database Analysis

PrognoScan database (http://www.abren.net/PrognoScan/) is an online tool for searching relationships between gene expression level and prognostic value analyzed from a large collection of publicly available cancer microarray datasets 23. The correlation between E2F2 expression and prognosis in colorectal cancer patients was analyzed by using the PrognoScan database, such as overall survival (OS), and disease-specific survival (DSS) and disease-free survival (DFS). The significant threshold was set to a Cox P-value < 0.05.

### Enrichment analysis of GSEA

Gene set enrichment analysis (GSEA) was used in the present study, which can evaluate whether a previously defined set of genes has statistically significant and consistent differences between two biological states 24. The tumor samples were divided into low and high level of E2F2 groups according to the data downloaded from TCGA database. The R package clusterProfiler (version 3.6.0) was applied to conduct GSEA between low- and high- E2F2 groups 25. GSEA was run with MSigDB Collections of c2 (c2.all.v7.2.symbols.gmt). Adjusted p value < 0.05 and q-value (false discovery rate, FDR) < 0.25 were considered significantly enriched.

### Immune infiltration analysis

The gene markers for 24 immune cells were derived from a previous study 26. The level of tumor immune infiltration was identified using single-sample GSEA (ssGSEA) method with GSVA R package based on TCGA-COADREAD data sets. The correlation analysis between E2F2 and these 24 immune cell types was calculated by Spearman correlation test. Graphs and figures were generated using the ggplot2 R package.

### Statistically analysis

Bioinformatics analysis was performed in R version 3.6.3. The different expression of E2F2 between normal and tumor tissues was analyzed using the Wilcoxon signed-rank test and one-way ANOVA. The Fisher exact test, chi-square test, Wilcoxon signed-rank test, and logistic regression were used to estimate the correlation of E2F2 expression and clinicopathologic features. Receiver operator characteristic (ROC) curve analysis was applied, with the area under curve (AUC) used as index of diagnostic accuracy. In addition, we used the Kaplan-Meier method and Cox regression to evaluate the role of E2F2 expression in prognosis. In Cox regression analysis, statistically significant variables in univariate Cox regression were furtherly enrolled into multivariate Cox regression. P < 0.05 was accepted as statistically significant.

## Results

### Transcriptional levels of E2F2 in patients with CRC

**Figure 1:**
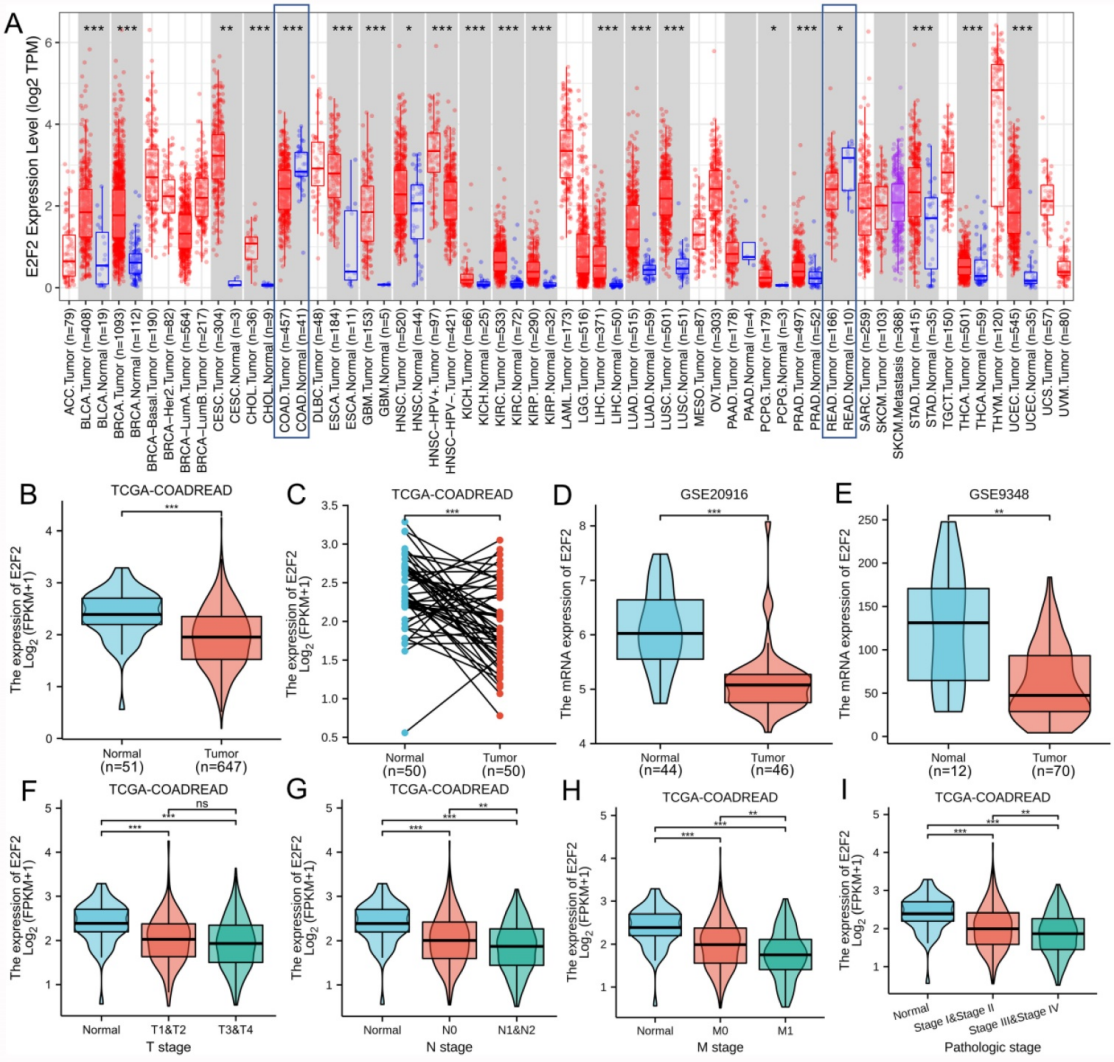
** Expression patterns of E2F2 mRNA in CRC from TIMER, TCGA and GEO database. (A)** The E2F2 expression in different cancer types from the TIMER database. **(B)** E2F2 mRNA expression was significantly downregulated in CRC tumor tissues compared to normal tissues from the TCGA-COADREAD data sets. **(C)** E2F2 mRNA expression was significantly decreased in paired CRC tumor tissues compared to adjacent normal tissues from the TCGA-COADREAD data sets. **(D)** E2F2 mRNA expression was significantly lower in colon adenocarcinoma than normal tissues from the GSE20916 dataset. **(E)** E2F2 mRNA expression was significantly reduced in early stage CRC tumor tissues compared to normal tissues from the GSE9348 dataset. **(F-I)** The mRNA expression level of E2F2 was analyzed using the TCGA-COADREAD data sets according to **(F)** T, **(G)** N, **(H)** M and **(I)** pathological stage. ns, no significant difference; * , *p* < 0.05; **, *p* < 0.01; ***, *p* < 0.001.

To determine the overall expression levels of E2F2 in different malignances, we firstly analyzed E2F2 expression across different cancer types using TIMER database. The results illustrated that E2F2 exhibited divergence in expression profiles among various cancers. The E2F2 mRNA expression in COAD (colon adenocarcinoma) and READ (rectum adenocarcinoma) was shown to be significantly downregulated compared with their normal tissues (**Figure 1A**). Furtherly, we explored the differentially expressed level of E2F2 in different cancer types using Oncomine database, which revealed that E2F2 mRNA expression was downregulated in colorectal cancer among three significant unique analyses (**Supplementary Figure S1**).

In order to furtherly determine the defferent expression level of E2F2 between colorectal tumor and normal tissues, we collected RNA-seq data and clinical information from 647 colorectal adenocarcinoma tissues and 51 colorectal normal tissues from the TCGA-COADREAD data sets. The results found that E2F2 was significantly downregulated in CRC tissues (p < 0.001, **Figure 1B**). Besides, we analyzed E2F2 expression level in 50 CRC tissues and their matched adjacent normal tissues, which showed that CRC tissues expressed lower E2F2 (p < 0.001, **Figure 1C**). In addition, to varify the above results, we downloaded the microarray data from GEO database, namely GSE20916. The results also illustrated that E2F2 was prominently downregulated in colon adenocarcinoma compared with nomal tissues (p < 0.001, **Figure 1D**). Moreover, GSE9348 data set was also downloaded to be analyzed and the results found that E2F2 was significantly decreased in tumor tissues of patients with early stage CRC compared with nomal tissues (p < 0.01, **Figure 1E**).

### Downexpressed E2F2 is correlated with poor clinicopathological features of colorectal cancer

The characteristics of 644 out of the 647 colorectal adenocarcinoma cases with complete clinical and gene expression data collected from TCGA datasets were shown in **Table 1**. According to E2F2 expression relative to the mean expression value, the patients with CRC were classified into high (n = 322) and low (n = 322) expression groups. Association between the E2F2 expression and different clinicopathological characteristics of CRC patients was evaluated and the results demonstrated that there was a significant correlation between the low E2F2 mRNA expression and higher N stage (p = 0.029), M stage (p = 0.002) and pathologic stage (p = 0.008). However, the associations between E2F2 mRNA expression and T stage, gender, age, CEA level, residual tumor, perineural invasion, lymphatic invasion, colon polyps present and tumor location were not statistically significant (all p > 0.05). In addition, similar patterns were found shown in **Figures 1F-I**, which revealed that low E2F2 expression was significantly correlated with N (N0 vs. N1/N2, p < 0.01, **Figure 1G**), M (M0 vs. M1, p < 0.01, **Figure 1H**) and pathology stage (stage I/stage II vs. stage III/stage IV, p < 0.01, **Figure 1I**). While, E2F2 mRNA expression showed no significant correlation with T stage (T1/T2 vs. T3/T4, p > 0.05, **Figure 1F**). Furthermore, univariate logistic regression analysis (**Table 2**) was also performed indicating that E2F2 mRNA expression was closely associated with N stage (OR = 0.727, 95% confidence interval (CI): 0.531-0.996, p = 0.047), M stage (OR = 0.465, 95% confidence interval (CI): 0.287-0.740, p = 0.001) and pathologic stage (OR = 0.714, 95% confidence interval (CI): 0.519-0.980, p = 0.038).

### Verification of E2F2 down-expression in CRC tumor tissues compared to normal tissues

**Figure 2:**
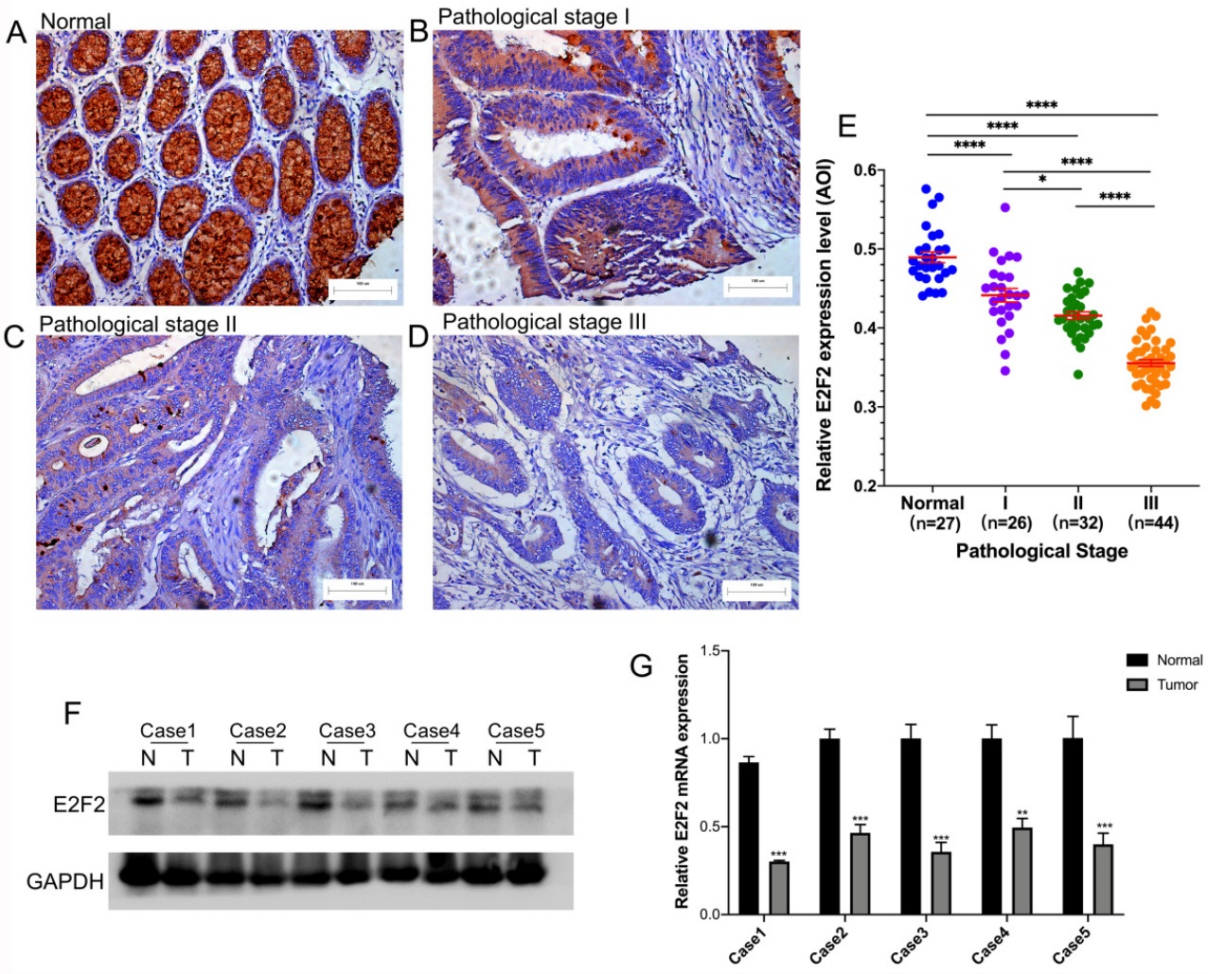
** E2F2 expression level was reduced in clinical CRC tissues. (A-D)** Representative images of E2F2 protein expression level in paraffin-embedded (A) normal mucosa and CRC tissues with (B) pathological stage I, (C) pathological stage II and (D) pathological stage III. Scales represent 100 micron. Representative images with original magnificent at 200×. **(E)** Quantifications of the average optical density (AOD) for E2F2 protein expression in normal mucosa and CRC tissues with pathological stage I-III. **(F)** Western blotting analysis and **(G)** quantitative real-time polymerase chain reaction analysis of five paired samples of CRC tissues and adjacent normal tissues from randomly selected CRC patients. * , *p* < 0.05; **, *p* < 0.01; ***, *p* < 0.001; ****, *p* < 0.0001.

We carried out immunohistochemistry staining for E2F2 in 102 paraffin-embedded human CRC tumor tissues, in which 26 cases were at pathological stage I, 32 cases at stage II and 44 cases at stage III. In addition, 27 normal mucosa tissue samples were used as control. As shown in **Figures 2A-D,** positive E2F2 staining was located at the base of the colonic glands in normal mucosa. E2F2 was mainly detected in the cytoplasmic in the normal mucosa and CRC tumor tissues. The protein level of E2F2 was downregulated in CRC tissues compared with normal mucosa tissues. Remarkably, its expression level was negatively correlated to poor pathological stage (**Figure 2E**). In addition, the median value of E2F2 expression level in cancerous tissues was used as the cutoff point to divide the CRC patients into high (n = 51) or low (n = 51) subgroups. The association between E2F2 expression and various clinicopathological parameters were evaluated in our cohort (**Table 1**). The results showed that E2F2 protein expression levels were negatively correlated with T stage (p < 0.001), N stage (p <0.001), and pathologic stage (p < 0.001). While there was no significant correlation of E2F2 expression with patients' age, gender, CEA level, colon polyps present, tumor location, tumor differentiation and tumor size. These results were similar to the analyses in TCGA cohort, showing that low expression of E2F2 predicted the worse status of pathologic stages in patients with CRC.

The E2F2 expression level was furtherly examined by western blotting and RT-qPCR in 5 pairs of CRC tissues and their corresponding adjacent normal tissues, which were randomly selected. As shown in **Figure 2F-G**, E2F2 expression was significantly downregulated in the CRC tissues at both mRNA and protein levels, compared with adjacent normal tissues. The results above demonstrated that E2F2 could be involved in cancer initiation and progression for CRC patients.

### Low E2F2 expression predictes a poor prognosis in CRC patients

**Figure 3:**
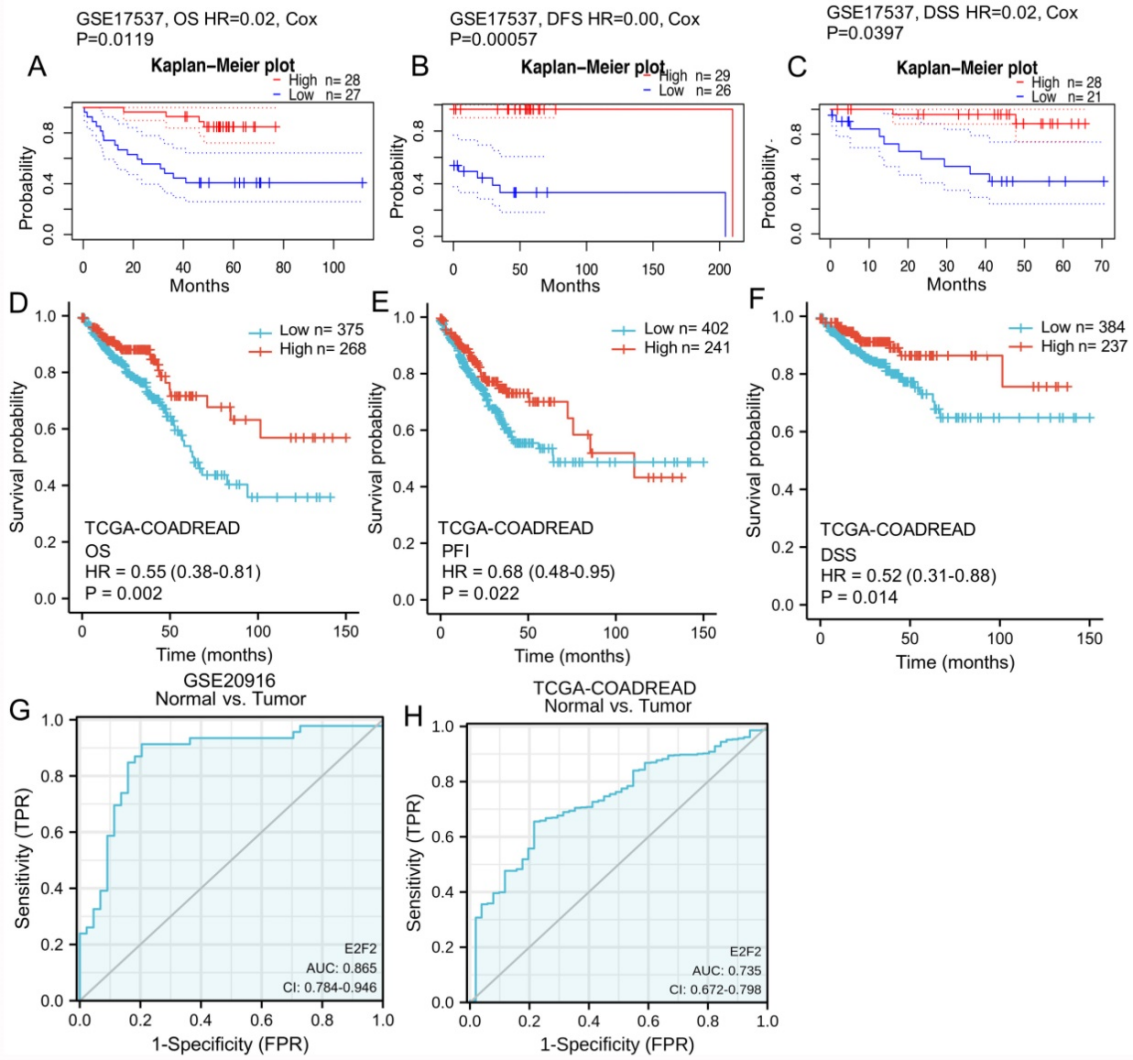
** The diagnostic and prognostic value of E2F2 in CRC. (A-C)** Kaplan-Meier survival curve analysis of OS, DFS and DSS showed that low E2F2 expression correlated to poor prognosis of CRC patients in a CRC cohort (GSE17537) from the PrognoScan database. **(D-F)** Survival curves showed OS, PFI and DSS rates of CRC patients with low or high E2F2 expression from the TCGA-COADREAD data sets. **(G, H)** ROC analysis illustrated that E2F2 expression accurately discriminated CRC tumor tissues from the normal tissues with an AUC of 0.865 (95% CI = 0.784-0.946) from GSE20916 data set and an AUC of 0.735 (95% CI = 0.672-0.798) from TCGA-COADREAD data sets. OS, overall survivial; DFS, disease free survival; DSS, disease specific survival; PFI, progress free interval; ROC: Receiver operating characteristic; AUC: area under the curve.

PrognoScan database was employed in order to investigate the role of E2F2 in survival rate of CRC patients. Two cohorts (GSE17536, GSE17537) 27, 28 composed of 177 samples and 56 samples at different stages of colon cancer were enrolled and analyzed, respectively. In GSE17536 cohort, low E2F2 expression was significantly correlated with poorer prognosis (DFS, HR = 0.23, 95% CI = 0.06 to 0.87, Cox P = 0.0303,** Supplementary Figure S1B**). In GSE17537 cohort, decreased E2F2 expression was also shown to be significantly associated with poorer prognosis (OS, HR = 0.02, 95% CI = 0.00 to 0.43, Cox P = 0.0119, **Figure 3A**; DFS, HR = 0.00, 95% CI = 0.00 to 0.08, Cox P = 0.00057,** Figure 3B**; DSS, HR = 0.02, 95% CI = 0.00 to 0.83, Cox P = 0.0397, **Figure 3C**). Therefore, the results suggested that down-regulated E2F2 expression is a risk factor for poor prognosis in CRC patients.

To furtherly varify the repeatability and portability of the correlative significance between the expression of E2F2 and prognosis, Kaplan-Meier survival analysis was conducted based on TCGA-COADREAD data sets. As shown in** Figures 3D-F**, low E2F2 expression was positively correlated with poor overall survival (OS) (HR = 0.55, 95% CI = 0.38-0.81, p = 0.002, **Figure 3D**). Similarly, we also observed that the decreased expression of E2F2 was significantly correlated with the poor progression-free interval (PFI) (HR = 0.68, 95% CI = 0.48-0.95, p = 0.022, **Figure 3E**) and disease-specific survival (DSS) (HR = 0.52, 95% CI = 0.31-0.88, p = 0.014, **Figure 3F**). In the univariate analysis, T stage, N stage, M stage, pathologic stage, age, E2F2 expression level and residual tumor affected the prognosis of CRC patients (all p < 0.05). Furtherly, multivariate Cox regression showed T stage, N stage, pathologic stage, E2F2 expression level, age and residual tumor were independent risk factors for unfavorable survival (OS) of CRC patients (**Table 3**).

Subgroup analysis was applied to show the impact of E2F2 expression in the progosis of CRC patients with high stage. As shown in **Figure S2**, low E2F2 expression had a significant relationship between poorer prognosis in stages T3 + T4, including OS (HR = 0.54, 95% CI = 0.36-0.82, p = 0.003, **Supplementary Figure S2A**), PFI (HR = 0.62, 95% CI = 0.43-0.88, p = 0.008, **Supplementary Figure S2B**) and DSS (HR = 0.47, 95% CI = 0.27-0.80, p = 0.006, **Supplementary Figure S2C**). In CRC patients with down-regulated E2F2 expression, the survival rates were also lower in stages N1 +N2, including OS (HR = 0.49, 95% CI = 0.29-0.83, p = 0.009, **Supplememtary Figure S2D**), PFI (HR = 0.59, 95% CI = 0.37-0.95, p = 0.028, **Supplememtary Figure S2E**) and DSS (HR = 0.47, 95% CI = 0.25-0.89, p = 0.02, **Supplememtary Figure S2F**). In addition, similar results were shown that CRC patients in pathological stages III + IV had poorer progosis with lower E2F2 expression, including OS (HR = 0.48, 95% CI = 0.28-0.83, p = 0.008, **Supplememtary Figure S2G**), PFI (HR = 0.59, 95% CI = 0.37-0.94, p = 0.025, **Supplememtary Figure S2H**) and DSS (HR = 0.45, 95% CI = 0.24-0.84, p = 0.013, **Supplememtary Figure S2I**). However, it had no statistical correlation between E2F2 mRNA expression and prognosis of CRC patients with T0 + T1 stage, N0 stage or pathological stage I + II stage, respectively (data not shown). The results revealed that E2F2 expression is a potential independent prognostic biomarker in CRC patients, especially in advanced stage.

### E2F2 expression is a potential diagnostic biomarker in CRC patients

ROC analysis was applied to evaluate the effectiveness of E2F2 mRNA expression level to distinguish colorectal adenocarcinoma from normal tissues, which estimated AUC at 0.865 (95% CI: 0.784-0.946, **Figure 3G**) in the GSE20916 data set. In TCGA-COADREAD data sets, to evaluate the potential value of E2F2 expression to distinguish CRC tissues from normal tissues, AUC of E2F2 was 0.735 (95% CI: 0.672-0.798, **Figure 3H**). In addition, ROC analysis of different subgroups of CRC patients elucidated that E2F2 expression lent a degree of credibility to distinguish early and advanced stage patients, including (T1-2) vs. (T3-4) stage (AUC = 0.538, 95% CI: 0.486-0.591, **Supplementary Figure S3A**), N0 vs. (N1-2) stage (AUC = 0.576, 95% CI: 0.531-0.621, **Supplementary Figure S3B**), M0 vs. M1 (AUC = 0.607, 95% CI: 0.543-0.671, **Supplementary Figure S3C**), pathology stage (I-II) vs. (III-IV) (AUC = 0.571, 95% CI: 0.526-0.617, **Supplementary Figure S3D**). The results above revealed that E2F2 could serve as a good diagnostic biomarker for CRC patients.

### Predicted biological function and pathways of E2F2 in CRC

**Figure 4:**
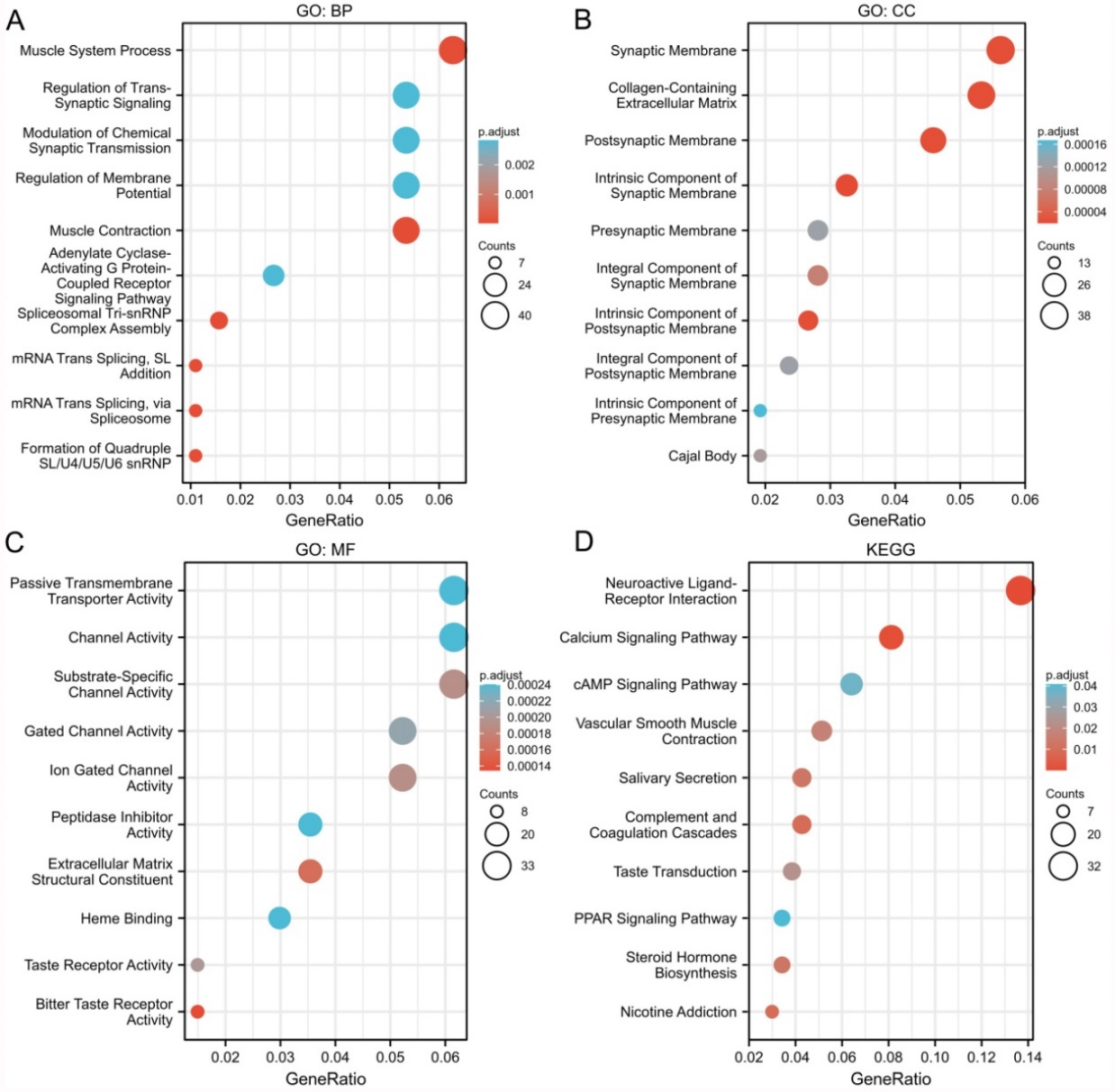
** Go and KEGG enrichment analysis of genes related to E2F2 in CRC tissues in the TCGA-COADREAD data sets. (A-C)** Go enrichment analysis showed the BP (biological processes), CC (cellular components), and MF (molecular function) of co-expressed genes with E2F2. **(D)** Significantly enriched KEGG terms obtained from KEGG enrichment analysis of co-expressed genes with E2F2.

Next, we analyzed the potential biological function, genes coexpressed with E2F2 (|logFC| > 1, P.adj < 0.05) were selected to perform gene enrichment analysis. GO term analysis for biological process (BP) showed that muscle system process, regulation of trans-synaptic signaling, modulation of chemical synaptic transmission and adenylate cyclase-activating G protein-coupled receptor signaling pathway were significantly enriched (**Figure 4A**). Go term analysis for cellular component (CC) showed that synaptic membrane, Collagen-containing extracellular matrix, postsynaptic membrane were mainly enriched (**Figure 4B**). The molecular fuction (MF) analysis showed that passive transmembrane transporter acitivity, channel acitivity and substrate-specific channel acitivity were significantly enriched (**Figure 4C**). KEGG analysis revealed that neuroactive ligand-receptor interaction was the most significantly enriched pathway (**Figure 4D**). Overall, the results indicated that E2F2 and its coexpressed genes may participate in cell signaling functions and the “neuroactive ligand-receptor interaction” pathway, which in turn regulate proliferation and invasion of CRC.

**Figure 5:**
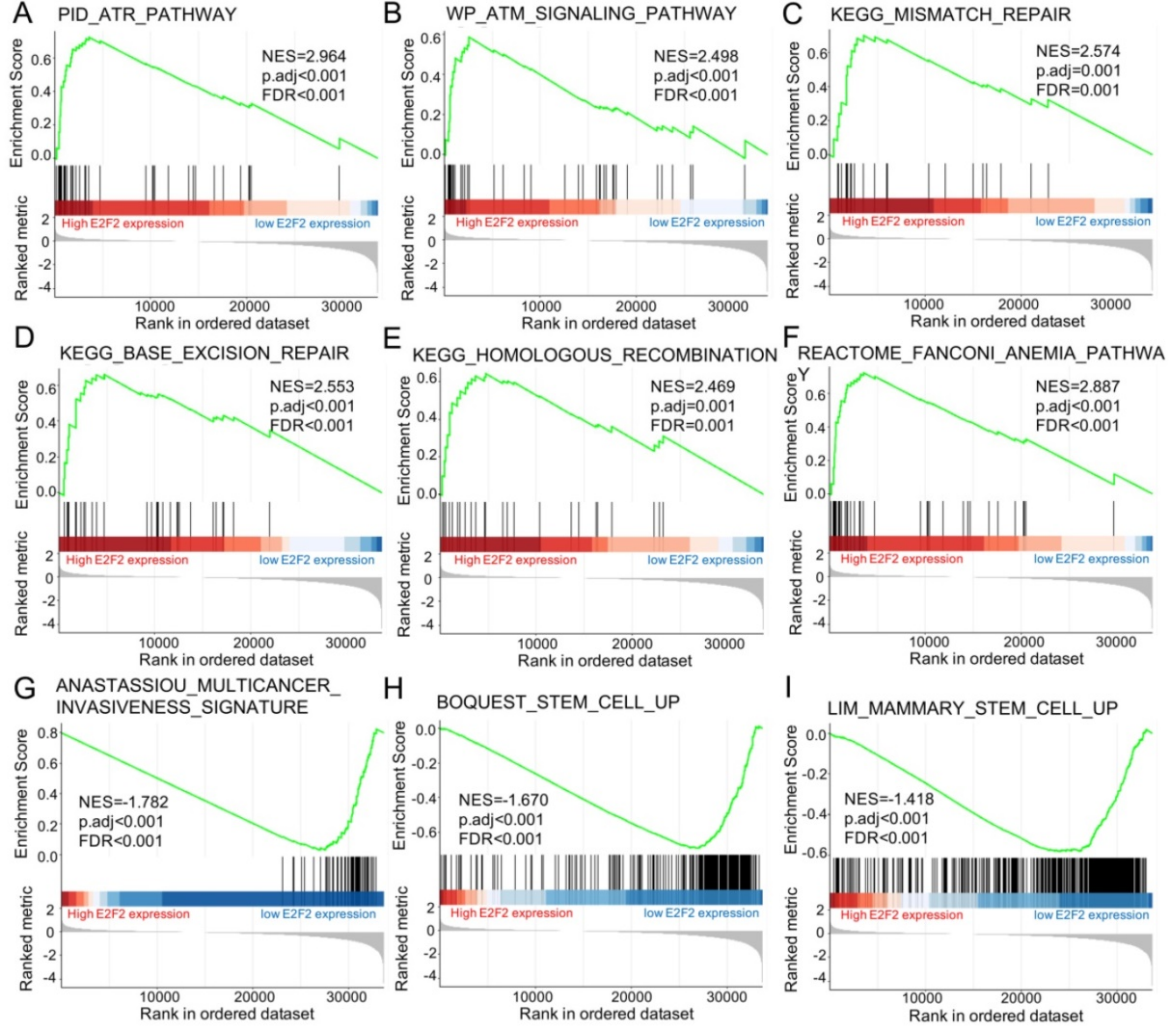
** Enrichment plots from the gene set enrichment analysis (GSEA). (A)** ATR pathway, **(B)** ATM signalling pathway, **(C)** mismatch repair, **(D)** base excision repair, **(E)** homologous recomibination, **(F)** Fanconi Anemia pathway, **(G)** multicancer invasiveness signature, **(H)** stem cell up, and** (I)** mammary stem cell up were significantly enriched in E2F2-related CRC. NES, normalized enrichment scores; FDR, false discovery rate.

Furtherly, GSEA was conducted to elucidate the possible biological pathways regulated by E2F2 between high and low E2F2 expression groups based on the nomalized enrichment score (NES) and FDR (false discovery rate) q-value. As shown in **Figure 5** and** Table 4**, several signal pathways were significantly enriched in high E2F2 expression group, including ATR pathway (**Figure 5A**), ATM signalling pathway (**Figure 5B**), mismatch repair (**Figure 5C**), base excision repair (**Figure 5D**), homologous recomibination (**Figure 5E**) and Fanconi Anemia pathway (**Figure 5F**). Moreover, multicancer invasiveness signature (**Figure 5G**) and cancer stem cells (**Figure 5H-I**) were significantly enriched in low E2F2 expression group.

### Correlations of E2F2 expression and immune infiltration level in CRC

**Figure 6:**
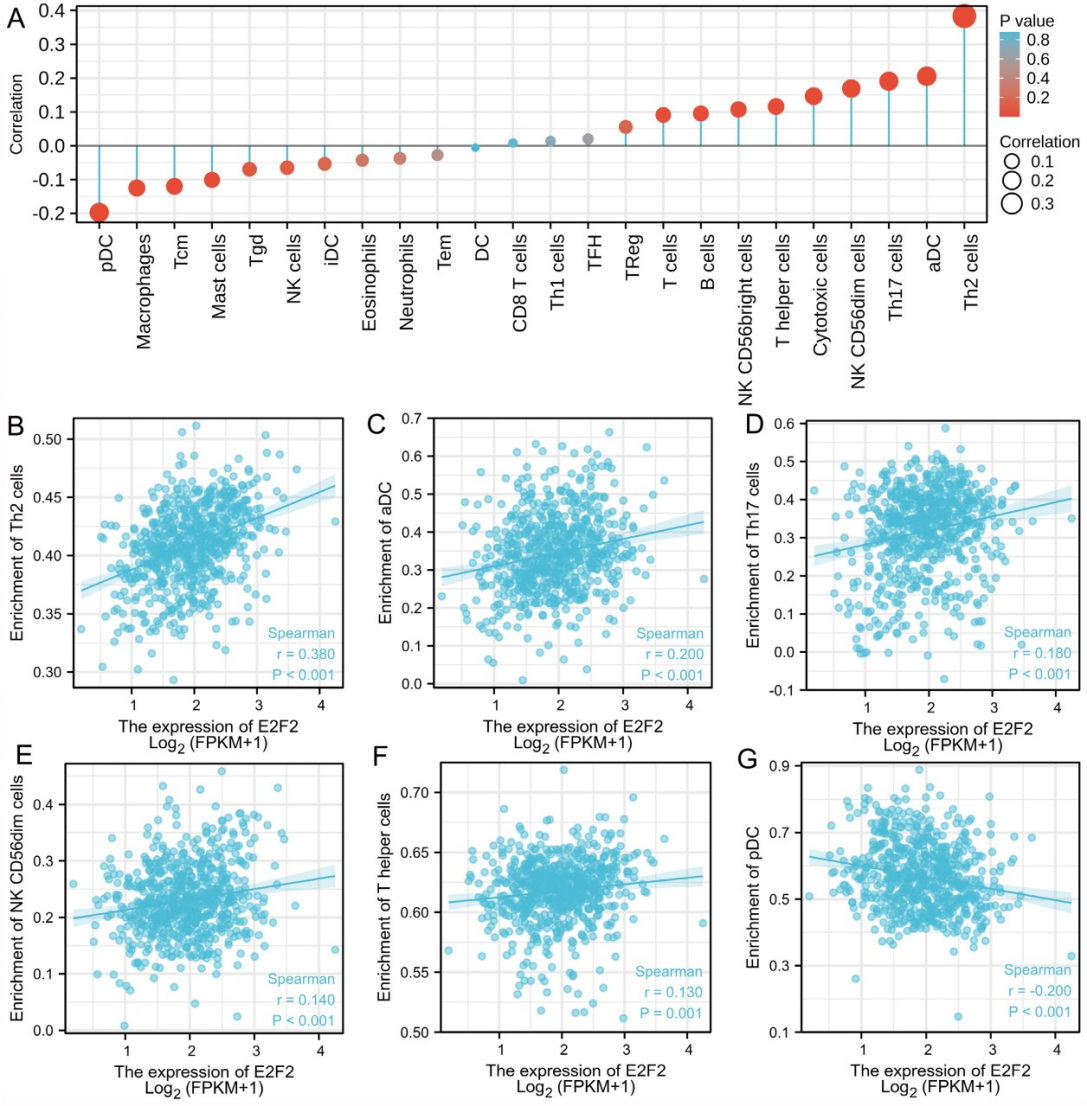
** Association analysis of E2F2 gene expression and immune infiltration. (A)** The association between E2F2 expression and 24 tumor-infiltrating lymphocytes. **(B-G)** The correlation of E2F2 expression with immune infiltration level of **(B)** Th2 cells, **(C)** aDC cells, **(D)** Th17 cells, **(E)** NK CD56dim cells, **(F)** T helper cells, **(G)** pDC cells.

As reported, the tumor-infiltrating lymphocytes correspond to improved prognosis in various cancers 26, 29. Therefore, we furtherly explored the association between E2F2 expression and immune infiltration in CRC. Through analyzing the correlation between E2F2 and 24 immune-cell subsets in CRC by means of ssGSEA with Spearman r (**Figure 6A**), we found that E2F2 was positively correlated with infiltration levels of Th2 cells (R = 0.380, p < 0.001, **Figure 6B**), aDC (R = 0.200, p < 0.001, **Figure 6C**), Th17 cells (R = 0.180, P < 0.001, **Figure 6D**), NK CD56dim cells (R = 0.140, p < 0.001,** Figure 6E**), T helper cells (R = 0.130, p = 0.001,** Figure 6F**), cytotoxic cells (R = 0.120, p = 0.002, data not shown) and B cells (R = 0.089, p = 0.023, data not shown). Moreover, E2F2 expression was negatively associated with infiltration level of pDC (R = -0.200, p < 0.001, **Figure 6G**), macrophages (R = -0.110, p = 0.006, data not shown), Tcm (R = -0.081, p = 0.04, data not shown), mast cells (R = -0.091, p = 0.020, data not shown), Tgd (R = -0.089, p = 0.023, data not shown), NK cells (R = -0.130, p = 0.001, data not shown). This results unveiled that E2F2 potentially regulates lymphocytes infiltration in the tumor of CRC.

## Discussion

Due to the insidious early symptoms and the shortage of effective early-diagnostic methods, most newly diagnosed patients with CRC are already at the advanced stage, which usually present with synchronous metastasis, resulting in a poor prognosis 30, 31. Currently, traditional biomarkers, carbohydrate atigen19-9 (CA19-9) and carcinoembryonic antigen (CEA) play an unsatisfactory role in detection of CRC due to its low sensitivity 32, which reinforces the importance of determining better biomarkers for early CRC screening and post-surgical relapse cases. Studies have revealed that inactivation of tumor-suppressor genes and activation of oncogenes are important causes in the pathogenesis of CRC 33. Therefore, there is an urgent need for identifing effective prognostic biomarkers and cancer-related molecular mechanisms for discovering therapeutic targets in CRC.

The E2F family of transcription factors is the downstream of the cyclin-dependent kinase (CDK)-RB-E2F network, whose protein pruducts form the core transcriptioanal machinery involving in cell cycle progression 34. More recent studies have shown that dysregulated E2Fs also have roles in mediating mutiple hallmarks of cancer, including DNA damage response, genomic stabiligy, apoptosis, angiogenensis and metabolism 11, 34. The function of the E2F transcription factors is complex, which could serve as tumor suppressors or oncogenes depending on the context or environment of the interaction 34. In the E2F family, E2F1 is the most extensively investigated member across different types of cancer. In CRC, previous studies have reported 35-37 that elevated E2F1 expression exhibits an inverse association with cell proliferation and a positive correlation with increased apoptotic levels, suggesting a tumor-suppressive role in CRC progression. However, recent evidence points to a tumor-promoting role of E2F1 in CRC. For example, E2F1 was reported to promote tumor growth, invasion and metastasis of CRC cells by activating the ribonucleotide reductase small subunit M2 (RRM2) transcription 38. SiRNA-mediated E2F1 knockdown can significantly suppress cell proliferation of CRC cell 39. Higher E2F1 expression in CRC tissues is significantly correlated to with poorer overall survival of patients with CRC 39.

E2F2, as an important member of E2F family, has attracted growing research attenttion in tumorigenensis and tumor progression in different malignancies. Previous researches have revealed that elevated E2F2 is significantly correlated with poor prognosis in hepatocellular carcinoma 40, ovarian cancer 14 and breast cancer 41. It has been discovered that E2F2, a direct target gene of Let-7b, can be used for targeted treatment to promote the proliferation and stem-like cell of glioma cells 15. Similar results provided evidence that miR-31 inhibits E2F2 expression to suppress tumor cell malignant phenotypes in gastric cancer 42. Additionally, E2F2 was also reported to promotes proliferation through inhibiting p53-dependent apoptosis pathway in human melanoma cells 16. E2F2 is regarded as an oncogene in several types of malignant tumors, however, it can also be designated as a tumor suppressor gene. Opavsky R, et al. 18 reported the specific tumor suppressor function of E2F2 in Myc-induced T cell lymphomagenesis. Besides, Yu Gao et al. 17 indicated that E2F2 is down-expressed in clear cell renal cell carcinoma cancer tissues and it represses cell proliferation and invasion, targeted by miR-155.

Although E2F2 has been well studied in multiple cancer types, it remains unclear in colorectal cancer. A study reported that nuclear immune-expression of E2F2 in CRC tumor cells is extremely low, and its nuclear immunoreactivity has no correlation with the kinetic parameters, leading to the hypothesis that E2F2 and E2F1 may exhibit functional redundancy within themselves sharing similar functions 37. Herein, we performed a bioinformatic analysis of E2F by using multiple public datasets about gene expression profiles in patients with CRC, finding the significant downregulation of E2F2 in the cancer tissues of CRC patients, which was further experimentally confirmed *in vivo* with the CRC tumor tissues and normal mucosa tissue controls by using multiple assays including immunohistochemical staining, qRT-PCR and western blot.

In our present study, we found that E2F2 mRNA expression varied across different types of cancers. Then we investigated E2F2 expression in CRC based on TCGA and GEO databases and our clinical specimens, which illustrated that E2F2 expression was significantly downregulated in the CRC tissues at both mRNA and protein levels, compared with normal tissues. Remarkably, the expression level of E2F2 was negatively correlated with poor clinicopathological characteristics in CRC. Moreover, downregulation of E2F2 was statistically associated with poor OS, DSS, DFS and PFI in CRC. Most importantly, univariate and multivariate cox regression analysis both suggested that E2F2 was an independent prognostic factor for CRC patients. In addition, ROC analysis lent a high degree of credibility for the diagnostic value of E2F2 in patients with CRC.

To furtherly explore the potential role of E2F2 in CRC, TCGA data were used for GO and KEGG analysis of the E2F2-coexpressed genes and GSEA analysis for E2F2. In the GO analysis, biological processes related to cell signaling functions were identified, including regulation of trans-synaptic signaling, modulation of chemical synaptic transmission and adenylate cyclase-activating G protein-coupled receptor signaling pathway, etc. KEGG analysis revealed that neuroactive ligand-receptor interaction was the most significantly enriched pathway. Neuroactive ligand-receptor interaction has been considered to participate in tumorigenesis and development in many cancer types, such as breast cancer 43, renal cell carcinoma 44, glioma 45, and hepatocellular carcinoma 46. E2F2 and its coexpressed genes may be involved in cell signaling functions and the “neuroactive ligand-receptor interaction” pathway, which in turn are required for cancer initiation and progression of CRC.

In GSEA analysis, several pathways were significantly enriched corresponding to the E2F2 high expression phenotype, including ATR pathway, ATM signalling pathway, mismatch repair, base excision repair, homologous recomibination and Fanconi Anemia pathway. These pathways are closely related to DNA damage repair and genome stability maintenance in CRC 47-50. A previous study has reported that E2F2 transcriptionally accumulates in response to DNA damage, promotes Rad51 foci formation and maintains the genomic stability and integrity in neuronal cells 51. More recently, it was reported that low E2F2 activity is correlated with high genomic instability and poor response to PARPi 52. Notebally, it has been poorly described for the role of E2F2 in gene instability and repair in the context of cancer until now. Our present study firstly reported the potential role of E2F2 in regulating of DNA damage repair and mantainess of genomic instability in CRC. In addition, multicancer invasiveness signature and stem cell genes were significantly enriched in the E2F2 low expression phenotype, which indicated that E2F2 may play a role in modulating cancer invasion and cancer stem cells in CRC.

Furthermore, to depict the immune infiltration level in CRC, we evaluated the association between E2F2 and immune cell populations based on transcriptomic data. The results illustrated that E2F2 expression had extensively correlation with immune infiltrates, inculding Th2 cells, T17 cells, T helper cells, Tgd, Tcm, aDC, pDC, NK cells, NK CD56dim cells, cytotoxic cells, B cells, macrophages and mast cells, and these cells play a critical role in cancer control. The immune microenvironment of tumor cells and immune-related mechanisms contribute to the development of tumors and efficiency of cancer therapy, and closely relate to clinical outcomes 26, 29. Data from our present study provide evidence that E2F2 participates in regulating immune infiltration in the local microenvironment of CRC. However, unbiased methods are required to furtherly analyze the functions and pathways of E2F2 in tumor immune infiltration in CRC.

The importance and originality of our analysis in the present study were that it provided one of the first systematic investigations into the relationship between E2F2 and CRC. However, there were still some limitations. Firstly, our study relied mainly on bioinformatic analyses, further experimental researches* in vitro* and *in vivo* should be required in our laboratory. Secondly, the number of databases used in our study were limited, thus, we should cross validate our results in multiple datasets. Last but not least, our study had the inherent limitations of a retrospective study design. Therefore, prospective studies with a large sample size are needed to confirm our findings.

## Conclusions

In conclusion, E2F2 is downregulated in CRC tissues compared to their matched adjacent normal tissues. Low expression of E2F2 is closely correlated to the advanced pathological stage and poor prognosis, including OS, DSS, DFS and PFI. ROC analyses partially unveil that E2F2 can serve as a good predictive biomarker for discriminating colorectal adenocarcinoma and normal tissue. Moreover, E2F2 is possibly involved in CRC tumorigenesis and development via modulating ATR pathway, ATM signalling pathway, mismatch repair, base excision repair, homologous recomibination, Fanconi Anemia pathway, multicancer invasiveness signature, cancer stem cells and immune infiltrating cells. Further experimental researches *in vitro* and *in vivo* are required to confirm our findings.

## Supplementary Material

Supplementary figures.

## Figures and Tables

**Table 1:** Association of E2F2 expression and clinicopathological parameters in patients with CRC

Characteristics	E2F2 expression in TCGA cohort			E2F2 expression in our cohort		
	Low (n = 322)	High (n = 322)	*p*	Low (n = 51)	High (n = 51)	*p*
**T stage, n (%)**			0.198			**< 0.001**
T1	10 (1.6%)	10 (1.6%)		0 (0%)	3 (2.9%)	
T2	48 (7.5%)	63 (9.8%)		5 (4.9%)	19 (18.6%)	
T3	217(33.9%)	219 (34.2%)		46 (45.1%)	29 (28.4%)	
T4	44 (6.9%)	30 (4.7%)				
**N stage, n (%)**			**0.029**			**< 0.001**
N0	171(26.7%)	197 (30.8%)		11 (10.8%)	47 (46.1%)	
N1	76 (11.9%)	77 (12%)		33 (32.4%)	3 (2.9%)	
N2	72 (11.2%)	47 (7.3%)		7 (6.9%)	1 (1%)	
**M stage, n (%)**			**0.002**			
M0	221(39.2%)	254 (45%)				
M1	58 (10.3%)	31 (5.5%)				
**Pathologic stage, n (%)**			**0.008**			**< 0.001**
Stage I	49 (7.9%)	62 (10%)		4 (3.9%)	22 (21.6%)	
Stage II	114(18.3%)	124 (19.9%)		7 (6.9%)	25 (24.5%)	
Stage III	91 (14.6%)	93 (14.9%)		40 (39.2%)	4 (3.9%)	
Stage IV	60 (9.6%)	30 (4.8%)				
**Gender, n (%)**			0.527			0.234
Female	155(24.1%)	146 (22.7%)		19 (18.6%)	21 (20.6%)	
Male	167(25.9%)	176 (27.3%)		32 (31.4%)	30 (29.4%)	
**Age, n (%)**			0.577			0.839
≤65	142 (22%)	134 (20.8%)		32 (31.4%)	30 (29.4%)	
>65	180 (28%)	188 (29.2%)		19 (18.6%)	21 (20.6%)	
**CEA level, n (%)**			0.28			0.539
≤5 ng/ml	125(30.1%)	136 (32.8%)		30 (29.4%)	34 (33.3%)	
>5 ng/ml	83 (20%)	71 (17.1%)		21 (20.6%)	17 (16.7%)	
**Residual tumor, n (%)**			0.516			
R0	226(44.3%)	242 (47.5%)				
R1	3 (0.6%)	3 (0.6%)				
R2	21 (4.1%)	15 (2.9%)				
**Perineural invasion, n (%)**			0.491			
No	100(42.6%)	75 (31.9%)				
Yes	38 (16.2%)	22 (9.4%)				
**Lymphatic invasion, n (%)**			0.722			
No	173(29.7%)	177 (30.4%)				
Yes	119(20.4%)	113 (19.4%)				
**Colon polyps present, n (%)**			1.000			0.820
No	126 (39%)	98 (30.3%)		37 (36.3%)	39 (38.2%)	
Yes	56 (17.3%)	43 (13.3%)		14 (13.7%)	12 (11.8%)	
**Location, n (%)**			0.787			0.549
Colon	241(37.4%)	237 (36.8%)		31 (30.4%)	27 (26.5%)	
Rectum	81 (12.6%)	85 (13.2%)		20 (19.6%)	24 (23.5%)	
**Differentiation, n (%)**						0.216
Well				1 (1%)	6 (5.9%)	
Moderate				48 (47.1%)	43 (42.2%)	
Poor				2 (2%)	2 (2%)	
**Tumor size, n (%)**						0.327
≤5 cm				38 (37.3%)	43 (42.2%)	
>5 cm				13 (12.7%)	8 (7.8%)	

Values shown in bold are statistically significant (P < 0.05); CRC, colorectal cancer; T: topography distribution; N: lymph node metastasis; M: distant metastasis; CEA: carcinoembryonic antigen.

**Table 2:** Logistic regression analysis of E2F2 expression associated with clinicopathological parameters in CRC

Characteristics	Total (N)	Odds Ratio (OR)	*p* value
T stage (T1/T2 vs. T3/T4)	641	0.758 (0.514-1.114)	0.159
N stage (N0 vs. N1/N2)	640	0.727 (0.531-0.996)	**0.047**
M stage (M0 vs. M1)	564	0.465 (0.287-0.740)	**0.001**
Pathologic stage (Stage I/Stage II vs. Stage III/Stage IV)	623	0.714 (0.519-0.980)	**0.038**
Gender (Female vs. male)	644	1.119 (0.821-1.526)	0.477
Age (>65 vs. ≤65)	644	1.107 (0.810-1.513)	0.524
CEA level (>5 vs. ≤5)	415	0.786 (0.527-1.171)	0.238
Residual tumor (R0 vs. R1/R2)	510	0.700 (0.366-1.320)	0.274
Perineural invasion (Yes vs. No)	235	0.772 (0.417-1.403)	0.401
Lymphatic invasion (Yes vs. No)	582	0.928 (0.666-1.294)	0.660
Colon polyps present (Yes vs. No)	323	0.987 (0.611-1.589)	0.958
Location (Colon vs. Rectum)	644	1.067 (0.749-1.520)	0.719

Values shown in bold are statistically significant (P < 0.05); CRC, colorectal cancer; T: topography distribution; N: lymph node metastasis; M: distant metastasis; CEA: carcinoembryonic antigen.

**Table 3:** Univariate and multivariate analysis of clinicopathological factors that correlate with OS of CRC patients

Characteristics	Total (N)	Univariate analysis		Multivariate analysis	
		Hazard ratio (95% CI)	p value	Hazard ratio (95% CI)	p value
T stage (T1/T2 vs. T3/T4)	640	2.468 (1.327-4.589)	**0.004**	3.377 (1.202-9.485)	**0.021**
N stage (N0 vs. N1/N2)	639	2.627 (1.831-3.769)	**<0.001**	0.287 (0.106-0.776)	**0.014**
M stage (M0 vs. M1)	563	3.989 (2.684-5.929)	**<0.001**	1.543 (0.808-2.948)	0.194
Pathologic stage (Stage I/II vs. Stage III/IV)	622	2.988 (2.042-4.372)	**<0.001**	7.222 (2.278-22.901)	**<0.001**
Gender (Female vs. Male)	643	1.054 (0.744-1.491)	0.769		
Age (>65 vs. ≤65)	643	1.939 (1.320-2.849)	**<0.001**	2.245 (1.341-3.759)	**0.002**
Residual tumor (R0 vs. R1/R2)	509	4.609 (2.804-7.577)	**<0.001**	2.197 (1.167-4.134)	**0.015**
Colon polyps present (Yes vs. No)	323	1.250 (0.743-2.103)	0.401		
Location (Colon vs. Rectum)	643	0.799 (0.519-1.230)	0.308		
E2F2 (Low vs. High)	643	0.689 (0.485-0.979)	**0.038**	0.570 (0.349-0.930)	**0.024**

Values shown in bold are statistically significant (P < 0.05); CRC, colorectal cancer; OS, overall survival; CI, confidence in interval; T: topography distribution; N: lymph node metastasis; M: distant metastasis.

**Table 4:** Results of gene set enrichment analysis (GSEA)

Description	Set Size	EnrichmentScore	NES	*p* value	*p*.adjust	q values	Rank
LIM_MAMMARY_STEM_CELL_UP	481	-0.581914	-1.41801	1.00E-10	2.95E-08	2.74E-08	9659
BOQUEST_STEM_CELL_UP	261	-0.6926715	-1.6698491	1.00E-10	2.95E-08	2.74E-08	6656
ANASTASSIOU_MULTICANCER_INVASIVENESS_SIGNATURE	64	-0.772308	-1.7822744	1.15E-10	3.14E-08	2.92E-08	6650
PID_ATR_PATHWAY	39	0.72454598	2.96436129	3.67E-10	9.11E-08	8.46E-08	3558
REACTOME_FANCONI_ANEMIA_PATHWAY	39	0.70570821	2.88728965	2.27E-09	4.47E-07	4.15E-07	5070
KEGG_BASE_EXCISION_REPAIR	33	0.65967707	2.55292218	5.49E-07	5.04E-05	4.68E-05	4725
WP_ATM_SIGNALING_PATHWAY	40	0.58657872	2.49812162	3.67E-06	0.00023251	0.00021594	2572
KEGG_MISMATCH_REPAIR	23	0.70023752	2.57439566	1.06E-05	0.00057049	0.00052983	3558
KEGG_HOMOLOGOUS_RECOMBINATION	28	0.64331955	2.4690956	1.08E-05	0.00057049	0.00052983	4737

## Abbreviations

E2F2: E2F family of transcription factor 2
CRC: colorectal cancer
COAD: colon adenocarcinoma
READ: rectum adenocarcinoma
BLCA: bladder urothelial carcinoma
BRCA: breast invasive carcinoma
CESC: cervical and endocervical cancer
CHOL: cholangiocarcinoma
ESCA: esophageal carcinoma
GBM: glioblastoma multiforme
HNSC: head and neck cancer
KICH: kidney chromophobe
KIRC: kidney renal clear cell carcinoma
KIRP: kidney renal papillary cell carcinoma
LIHC: liver hepatocellular carcinoma
LUAD: lung adenocarcinoma
LUSC: lung squamous cell carcinoma
PCPG: pheochromocytoma and paraganglima
PRAD: prostate adenocarcinoma
STAD: stomach adenocarcinoma
THCA: thyroid carcinoma
UCEC: uterine corpus
TIMER: Tumor Immune Estimate Resource
TCGA: The Cancer Genome Atlas
GEO: Gene Expression Omnibus
IHC: Immunohistochemistry
ROC: Receiver operating characteristic
AUC: area under curve
OS: overall survivial
DFS: disease free survival
DSS: disease specific survival
PFI: progress free interval
GO: gene ontology
BP: biological process
CC: cellular component
MF: molecular function
GSEA: Gene Set Enrichment Analysis
NES: normalized enrichment score
FDR: false discovery rate
