# Supplementary material for: Decreased E2F2 Expression Correlates with Poor Prognosis and Immune Infiltrates in Patients with Colorectal Cancer
Source: J Cancer. 2022 Jan 1;13(2):653–68. doi: 10.7150/jca.61415 (PMC8771517; doi:10.7150/jca.61415)
Supplement: Supplementary file 1 — Supplementary figures. [file jcav13p0653s1.pdf]

Supplementary materials

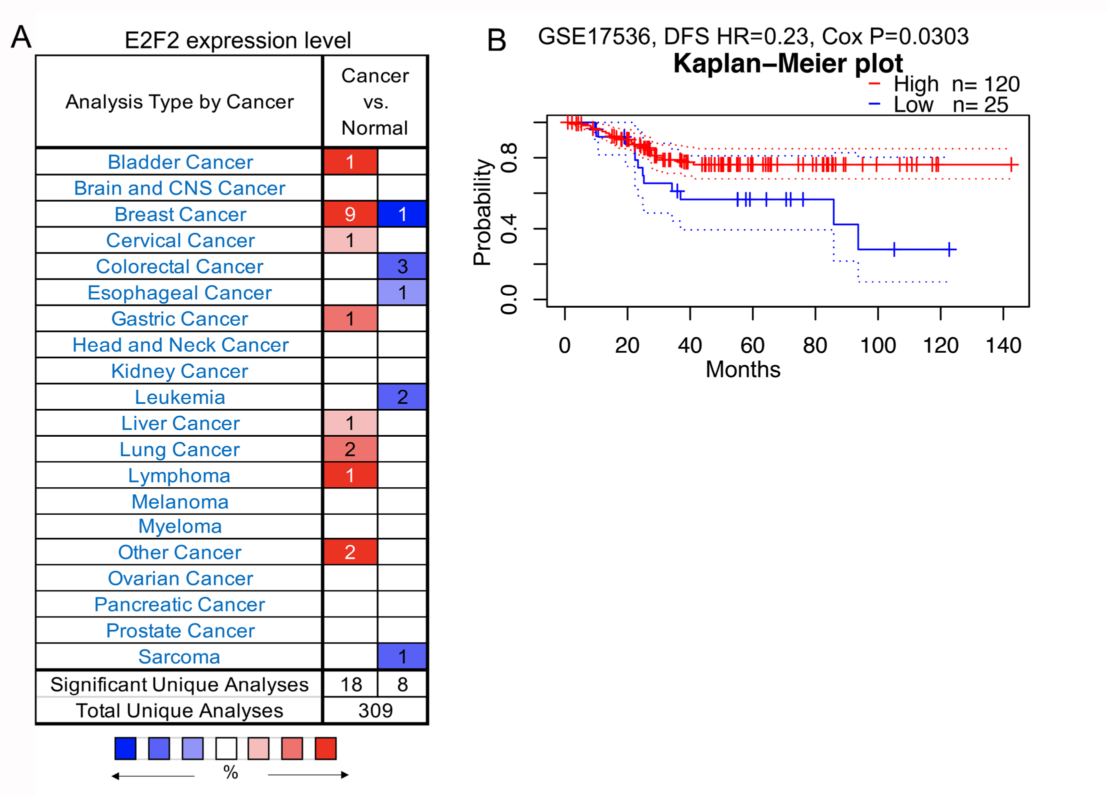

Figure S1. Expression patterns and the prognostic value of E2F2 in CRC. (A) E2F2 mRNA expression was downregulated in colorectal cancer among three significant unique analyses in oncomine database. Color: red, high expression; blue, low expression. (B) Survival curves of DFS in a CRC cohort (GSE17536) from the PrognoScan database. DFS, disease free survival

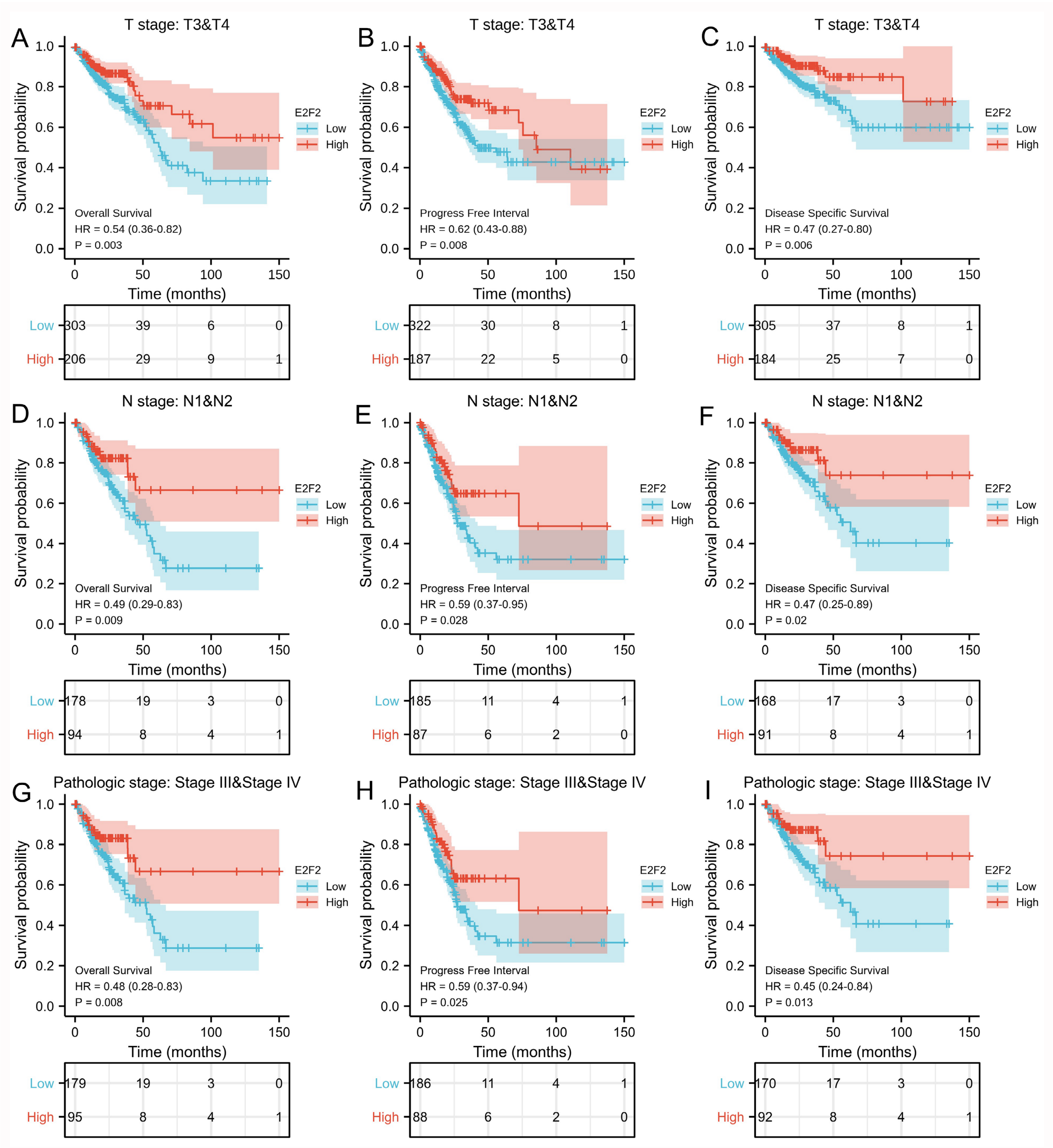

Figure S2. Kaplan-Meier survival curve analysis showed the correlation between E2F2 expression and prognosis in different subgroups of CRC patients from the TCGA-COADREAD data sets. (A-C) Survival curves of OS, PFI and DSS in CRC patients with T3+T4 stage. (D-F) Survival curves of OS, PFI and DSS in CRC patients with N1+N2 stage. (G-I) Survival curves of OS, PFI and DSS in CRC patients with pathology stage III+IV.

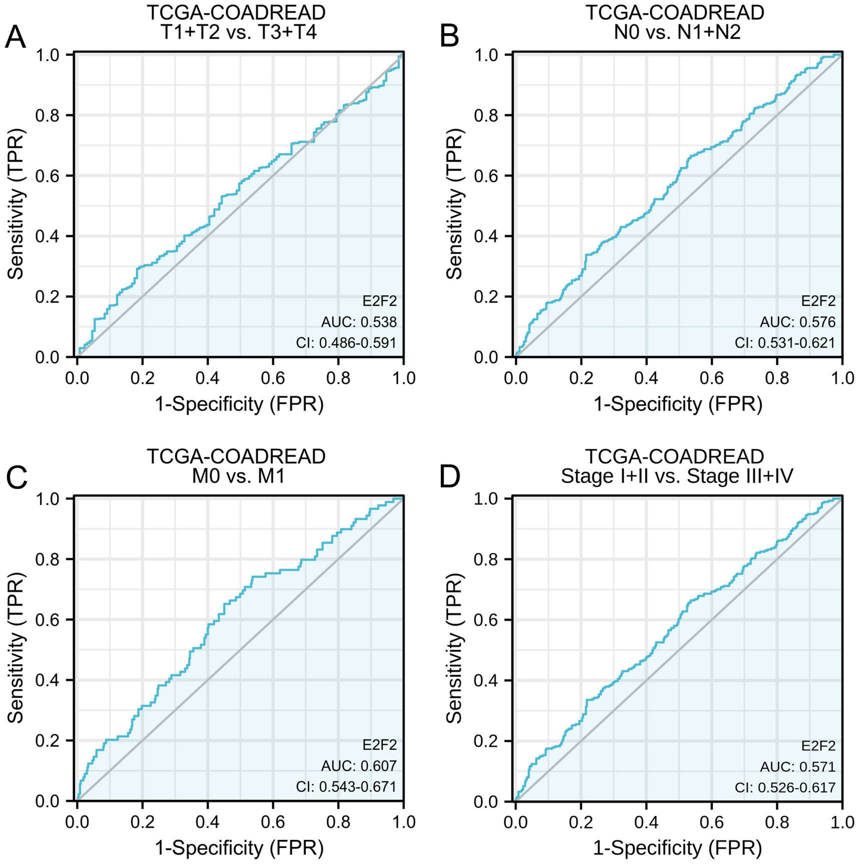

Figure S3. ROC analysis revealed that E2F2 expression had a diagnostic value to distinguish advanced- and early-stage CRC patients based on (A) T stage (T1+T2 vs. T3+T4), (B) N stage (N0 vs. N1+N2), (C) M stage (M0 vs. M1) and (D) pathology stage (Stage I+II vs. Stage III+IV).
